# Hybrid Theranostic Cubosomes for Efficient NIR-Induced Photodynamic Therapy

**DOI:** 10.1021/acsnano.1c09367

**Published:** 2022-03-25

**Authors:** Urszula Bazylińska, Dominika Wawrzyńczyk, Julita Kulbacka, Giacomo Picci, Livia Salvati Manni, Stephan Handschin, Marco Fornasier, Claudia Caltagirone, Raffaele Mezzenga, Sergio Murgia

**Affiliations:** † Department of Physical and Quantum Chemistry, Faculty of Chemistry, Wroclaw University of Science and Technology, Wybrzeze Wyspianskiego 27, 50-370 Wroclaw, Poland; ‡ Advanced Materials Engineering and Modelling Group, Faculty of Chemistry, Wroclaw University of Science and Technology, Wybrzeze Wyspianskiego 27, 50-370 Wroclaw, Poland; § Department of Molecular and Cellular Biology, Faculty of Pharmacy, Wroclaw Medical University, Borowska 211 A, 50-556 Wroclaw, Poland; ∥ Department of Chemical and Geological Sciences, University of Cagliari and CSGI, s.s. 554 bivio Sestu, I-09042 Monserrato, CA, Italy; ⊥ School of Medical Sciences, School of Chemistry and University of Sydney Nano Institute, The University of Sydney, Sydney, NSW 2006, Australia; ∇ ETH Zurich Department of Health Sciences & Technology, Schmelzbergstrasse 9, Zurich 8093, Switzerland; ¶ ETH Zurich Scientific Center for Optical and Electron Microscopy (ScopeM), Otto-Stern-Weg 3, Zurich 8093, Switzerland; # ETH Zurich Department of Materials, Wolfgang-Pauli-Strasse 10, Zurich 8093, Switzerland; ■ Department of Life and Environmental Sciences, University of Cagliari and CSGI, via Ospedale 72, I-09124 Cagliari, Italy; @ Department of Chemistry, Lund University, SE-22100 Lund, Sweden

**Keywords:** lipid bicontinuous cubic liquid-crystalline nanoparticles, up-converting nanocrystals, daunorubicin, layer-by-layer, MeWo cells, SKOV-3 cells, antitumor activity

## Abstract

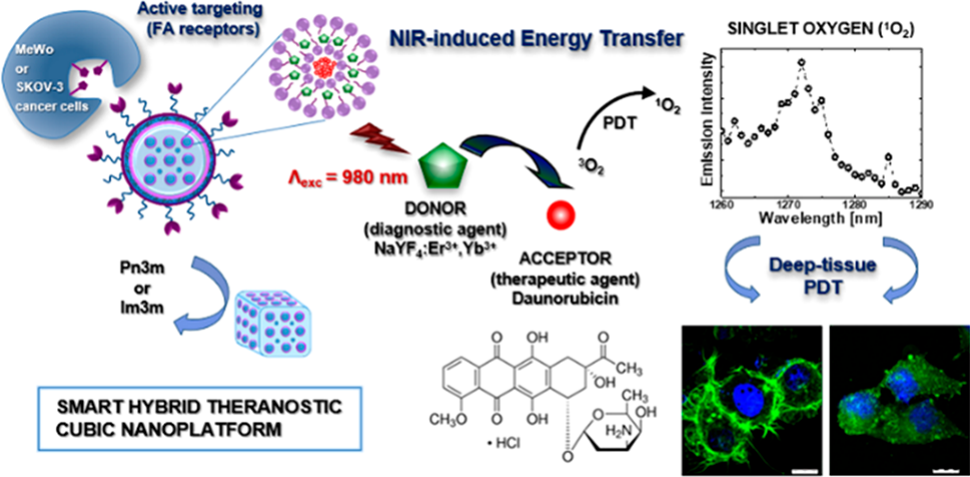

In recent years, lipid bicontinuous cubic liquid-crystalline nanoparticles known as cubosomes have been under investigation because of their favorable properties as drug nanocarriers useful for anticancer treatments. Herein, we present organic/inorganic hybrid, theranostic cubosomes stabilized in water with a shell of alternate layers of chitosan, single strand DNA (model genetic material for potential gene therapy), and folic acid–chitosan conjugate (the outmost layer), coencapsulating up-converting Er^3+^ and Yb^3+^ codoped NaYF_4_ nanoparticles and daunorubicin. The latter acts as a chemotherapeutic drug of photosensitizing activity, while up-converting nanoparticles serve as energy harvester and diagnostic agent. Cellular uptake and NIR-induced photodynamic therapy were evaluated *in vitro* against human skin melanoma (MeWo) and ovarian (SKOV-3) cancer cells. Results evidenced the preferential uptake of the theranostic cubosomes in SKOV-3 cells in comparison to uptake in MeWo cells, and this effect was enhanced by the folic acid functionalization of the cubosomes surface. Nanocarriers coloaded with the hybrid fluorophores exhibited a superior NIR-induced photodynamic activity, also confirmed by the improved mitochondrial activity and the most affecting f-actin fibers of cytoskeleton. Similar results, but with higher photocytotoxicity, were detected when folic acid-functionalized cubosomes were incubated with SKOV-3 cells. Taken on the whole, these results prove these hybrid cubosomes are good candidates for the photodynamic treatment of tumor lesions.

## Introduction

Over the past decade, research in drug delivery has been progressively focused toward redefining conventional therapeutic approaches into a more rationally designed model of treatment. Following this trend, combined delivery platforms have been developed, including colloidal self-assembled nanostructures characterized by a lipid phase as an essential prerequisite for specific functionality.^1−4^ These carriers, often endowed of theranostic modality, may be formulated in a different form, such as nanoemulsions, solid lipid nanoparticles, vesicular-type nanocarriers, or nonlamellar liquid-crystalline dispersions (hexosomes and cubosomes), and are designed to provide superior controlled intravenous or transdermal drug delivery, enhanced bioavailability, and specific targeting to pathological tissues followed by maximum therapeutic benefits, while simultaneously monitoring the progress of a given therapy.^3−6^ Among the aforementioned lipid nanocarriers, an important role in nanomedicine has been played by cubosomes,^7−9^ nanometric colloidal dispersion of bicontinuous cubic liquid-crystalline phases characterized by a honeycomb-like inner structure constituted by a lipid bilayer folded in space and separating two continuous, not interconnected water channels. Due to their peculiar nanostructure cubosomes are able to incorporate cargos with high loading efficiency^10−15^ and, given their potential as tools for traditional chemotherapy and diagnostics, gene therapy, and combined anticancer treatments by means of photodynamic therapy (PDT), definitely deserve to be included in the catalogue of the next generation of nanotheranostic candidates.

In addition, with proper cargo selection, cubosomes diagnostic and therapeutic functionalities may be controlled on-demand by NIR light external activation. For this purpose, inorganic *lanthanide-ion doped fluoride up-converting* nanocrystals can be used as energy harvesters and diagnostic tools, while organic chemotherapeutic dyes of photosensitizing ability may serve to improve the generation of reactive oxygen species (ROS) by NIR-activated static (inner filter effect or reabsorption) or dynamic (Förster or fluorescence resonance energy transfer – FRET) energy transfer.^16^ Indeed, lanthanide-ion doped fluoride up-converting nanoparticles (UCNPs) are particularly attractive for various biomedical applications and are now emerging as an interesting alternative for other diagnostic agents, including organic dyes or semiconductor quantum dots. The interest in biorelated applications of UCNPs is mainly due to their exceptional spectroscopic features, such as optical and chemical stability, lack of photobleaching, and long luminescence lifetimes.^17^ Owing to the character of 4f–4f transitions in lanthanide ions, there is an opportunity for NIR light absorption by, e.g., Yb^3+^ or Nd^3+^ ions and sequential energy transfer to neighbor ions such as Er^3+^, Tm^3+^, or Ho^3+^ ions that, in contrast to nonlinear absorption, does not require high power and femtosecond laser sources.^18^ As this up-conversion process is quite efficient for selected lanthanide ions pairs (e.g., Er^3+^/Yb^3+^, Tm^3+^/Yb^3+^, or Ho^3+^/Yb^3+^), it is possible to obtain visible luminescence after NIR excitation in the range 800–1300 nm (the so-called biological window) increasing the penetration depth and decreasing unwanted light absorption and scattering by biological chromophores (e.g., hemoglobin, oxyhemoglobin, or melanin).^19^ One of the particularly interesting and important biological applications of UCNPs is the possibility of NIR light harvesting and energy transfer to photosensitizer (PS) molecules, which may trigger the therapeutic effect of PDT.^19,20^ Since a large spectral overlapping between lanthanide emission lines and absorbance of PS molecules is required to facilitate the energy transfer, it deserves here noticing that the doping ions must be carefully selected.^21^ Thus, the approach involving the combination of the exceptional fluorescence activity of UCNPs with the therapeutic efficiency of commercially available PS (at sufficient spectral overlapping between UCNPs emission and PS absorbance spectra) can greatly enhance already existing PDT schemes. Moreover, designing efficient delivery carriers to address these cargos toward cancer cells represents a key point. Particularly, several methods for UCNPs and PS encapsulation within polymeric nanoparticles were presented,^22−24^ but formulation of cubosomes with these entities has not been considered yet.

Surface engineering can further extend functionality of cubosomes by a layer-by-layer (LbL) deposition of targeted and biocompatible polyelectrolytes (PEs) on the surface of such bicontinuous cubic liquid-crystalline nanoparticles. LbL structural design exploits the interface between the template and the solution as a specific site for selective adsorption of polyelectrolytes (including nucleic acids as DNA or RNA, and proteins) leading to multilayered nanostructures with precisely controlled thickness and permeability.^25−27^ The selection of an appropriate PE for the LbL deposition is crucial, since theranostic nanocarriers should be nontoxic and biocompatible while retaining physicochemical properties promoting specific interactions (such as cell adhesion and targeting ability) with pathological tissues.^26^ As it has been proved before, PEs of polysaccharide origin derived from naturally occurring chitins, alginates, or dextrans have appeared as the most attractive class of PEs since their sugar-based units are properly stable in the bloodstream and extracellular fluid and undergo rapid cleavage under intracellular reductive conditions, a desirable feature in drug delivery. Moreover, positively charged nanocontainers covered by an outer chitosan (CHIT) layer possess low hemolytic activity and improved cancer cell-adhesion properties, being at the same time competitive to common nanocarriers with PEG-ylated shell due to their low susceptibility to macrophage uptake.^27,28^ Thus, use of CHIT for the fabrication of a PE shell covering theranostic cubosomes could be profitable to extend the nanoparticle lifetime in the bloodstream and improve their biodistribution after intravenous administration. In addition, CHIT can be easily conjugated to targeting moieties for addressing cubosomes to pathological tissues. For example, folic acid (FA) molecules having selective affinity for folate receptors (FRs) may be useful in obtaining cubosomes with targeting response for the treatment of human carcinomas with overexpression of FRs, including ovarian, breast, and melanoma cancer cells.^29^

**Scheme 1:**
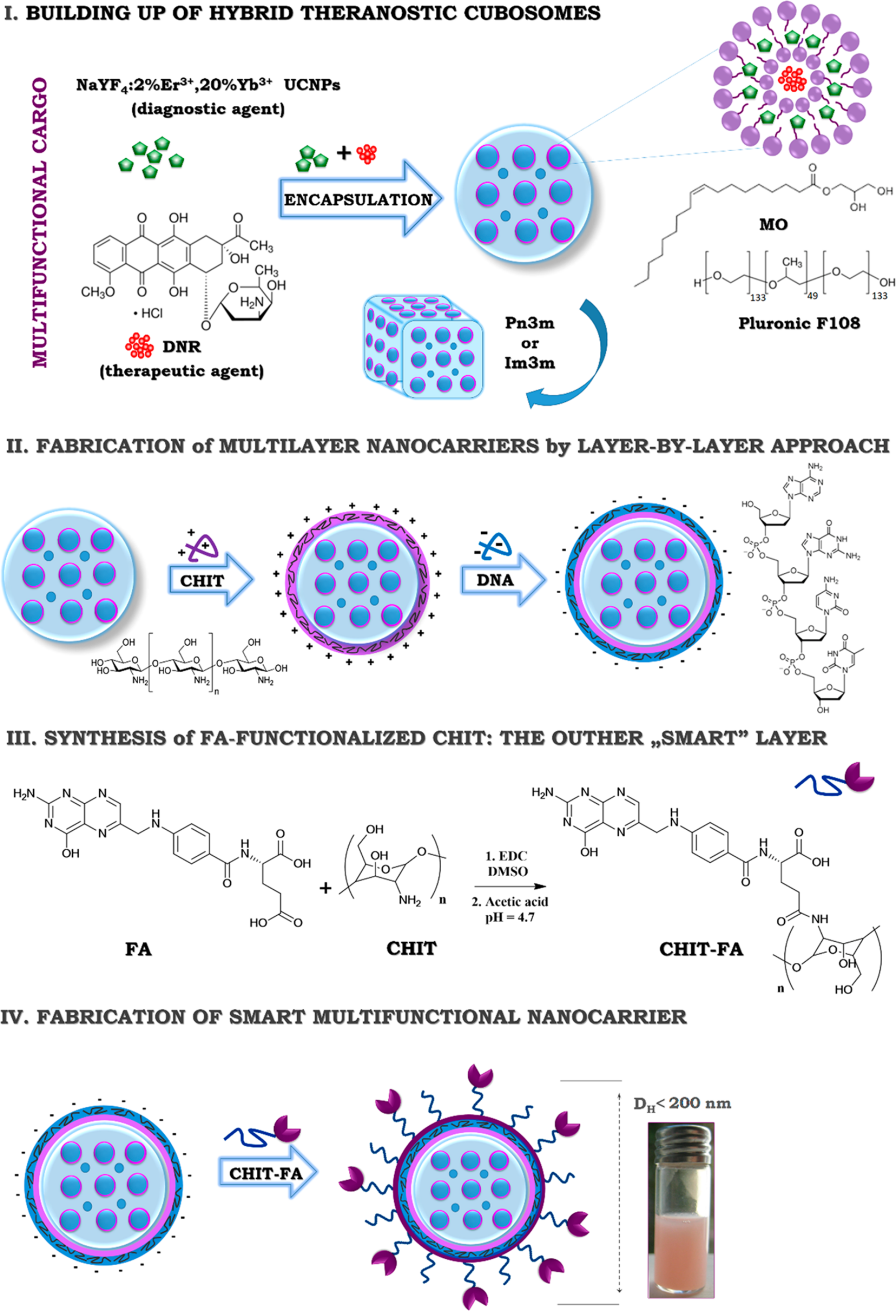
General Concept of the Performed Studies, with Individual Stages Leading to the Hybrid and Theranostic Bicontinuous Cubic Liquid-Crystalline Nanoparticles (Cubosomes)

In this work, an original hybrid, multifunctional, theranostic nanocarrier is engineered for anticancer applications by combining the exceptional fluorescence activity of NaYF_4_:2%Er^3+^,20%Yb^3+^ UCNPs and the therapeutic efficiency of commercially available photosensitive anticancer drug daunorubicin (DNR) with the peculiar delivery properties of cubosome nanoparticles covered by LbL with a folate–chitosan conjugate and a single strand DNA (of herring sperm origin) as model genetic material for potential gene therapy (Scheme 1).

## Results and Discussion

### Physicochemical Issues of Cubosomes with Optically Activatable Payloads

The simultaneous administration of multiple pharmacologically active and imaging agents may represent an efficient strategy to enhance the efficacy of a given therapy. However, coencapsulation of cargos within the same nanocarrier, also showing the proper colloidal stability for drug delivery applications, could be a main challenge. For this task, hydrophobic inorganic NaYF_4_:Er^3+^,Yb^3+^ UCNPs and hydrophilic organic DNR were here encapsulated within the bicontinuous cubic liquid-crystalline dispersion known as cubosomes formulation. The versatility of the formulation, initially stabilized in water by Pluronic F108, was further extended by LbL deposition of the biocompatible PEs CHIT and DNA on the surface of cubosomes. The LbL approach was performed by subsequent adsorption of the of CHIT/DNA PEs, from their solutions without the intermediate rinsing step, according to the procedure described in earlier studies related to other soft colloidal templates.^22,23^ Finally, folic acid-conjugated chitosan (FA-CHIT) was employed to produce multifunctional theranostic nanocarriers bearing targeting properties to cancer cells (such as ovarian, breast or melanoma) with overexpression of FRs.

**Figure 1:**
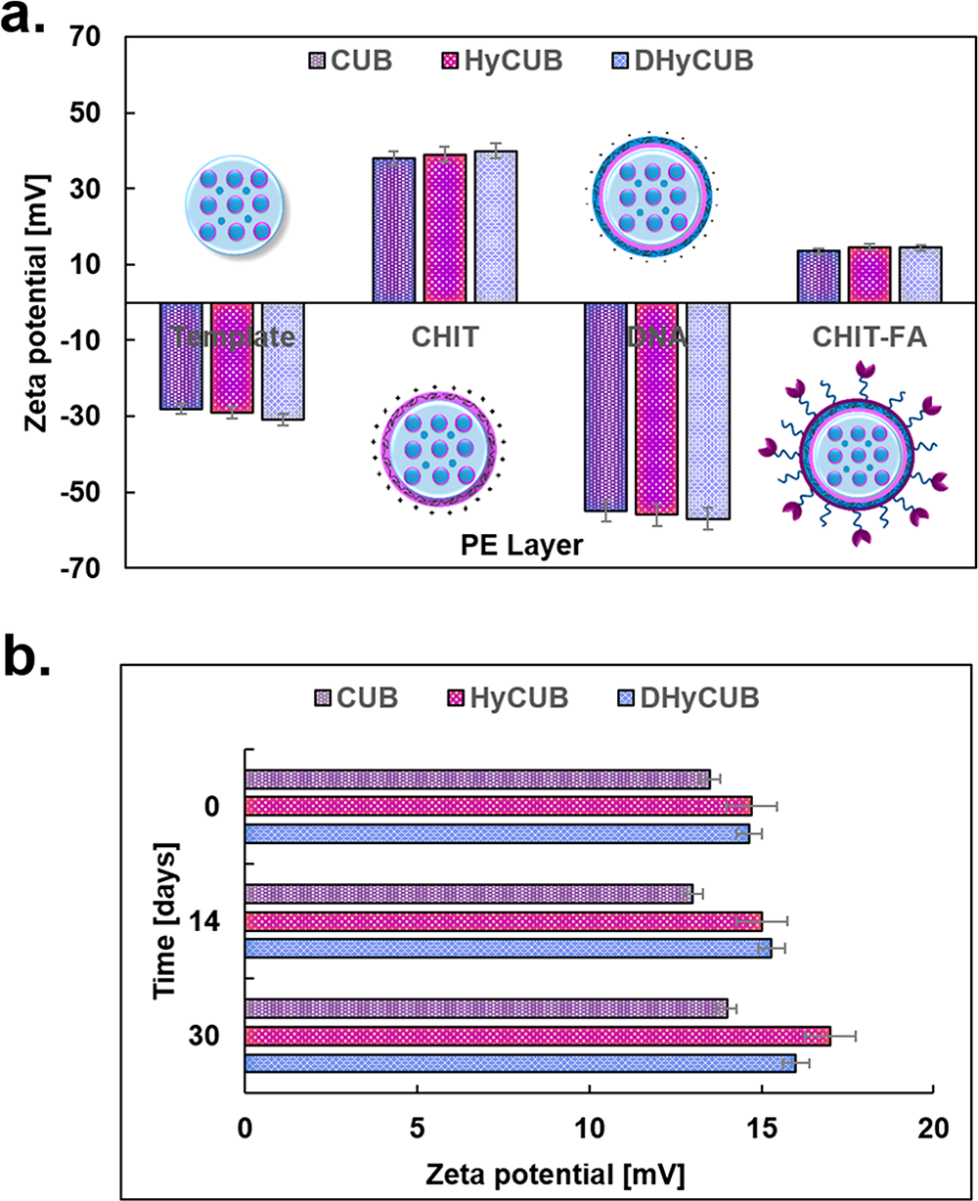
ζ-potential changes upon the CHIT/DNA/CHIT-FA layers deposition (a) and time-dependent colloidal stability of the obtained cubosomes (b).

The mean hydrodynamic diameter (*D*_H_), the polydispersity index (PdI), and the ζ-potential of empty cubosomes (CUB) and cubosomes loaded with UCNPs (HyCUB) or DNR+UCPNs (DHyCUB), with or without (sample names marked with an asterisk) the outermost layer of FA-CHIT, were evaluated by means of DLS and ELS (Table 1). As can be observed, the size of the cubosomes (between 154 and 169 nm) is only slightly affected by the presence of the cargos or by their covering with FA-CHIT. Differently, PdI values (in the range 0.163–0.254) show a marked increase when UCNPs are encapsulated in the cubosomes. However, size and PdI values did not deviate significantly from data previously reported for cubosome formulations.^6^ It deserves noticing that the external chitosan layer switches the usually observed negative ζ-potential of traditional cubosomes to positive values, proving the effective CHIT or FA-CHIT deposition on thecubosomes’ surface. Particularly, cubosome formulations coated by CHIT showed ζ-potential values around +40 mV, while values around +14 mV were recorded when FA-CHIT was used as the outermost layer. Figure 1a shows the typical changes of the ζ-potential upon adsorption of the consecutive PEs layer on the loaded or empty colloidal core.^26^ The detected regular layer to layer variations of CHIT/DNA ζ-potential from +38 to −55 mV for empty nanocarriers and from +40 to −57 mV for the loaded ones proved that stable PEs shells were obtained, and only an insignificant effect of the different payloads on the final ζ-potential values was observed. The stability test, showing minor variations of the ζ-potential values after storage in the dark (*t* = 30 days), proved the good colloidal stability of all the studied samples (Figure 1b).

**Table 1:** Hydrodynamic Diameter (*D*_H_), Polydispersity Index (PdI), and ζ-Potential of the Samples after LbL Deposition^a^

sample	*D*_H_/nm	PdI	ζ/mV
CUB	162 ± 7	0.184 ± 0.010	+13.5 ± 0.7
CUB*	154 ± 6	0.163 ± 0.010	+40.4 ± 2.0
HyCUB	167 ± 8	0.215 ± 0.012	+14.6 ± 0.8
HyCUB*	156 ± 6	0.254 ± 0.013	+38.4 ± 2.0
DHyCUB	169 ± 8	0.253 ± 0.013	+14.4 ± 0.7
DHyCUB*	163 ± 7	0.250 ± 0.013	+38.7 ± 2.0

a CUB: cubosomes. HyCUB: hybrid cubosomes loaded with up-converting nanoparticles. DHyCUB: hybrid cubosomes loaded with up-converting nanoparticles and daunorubicin. The asterisk indicates cubosomes without the outermost layer of folic acid-functionalized chitosan. *D*_H_: hydrodynamic diameter measured by DLS. PdI: polidyspersity index measured by DLS. ζ: zeta potential.

**Figure 2:**
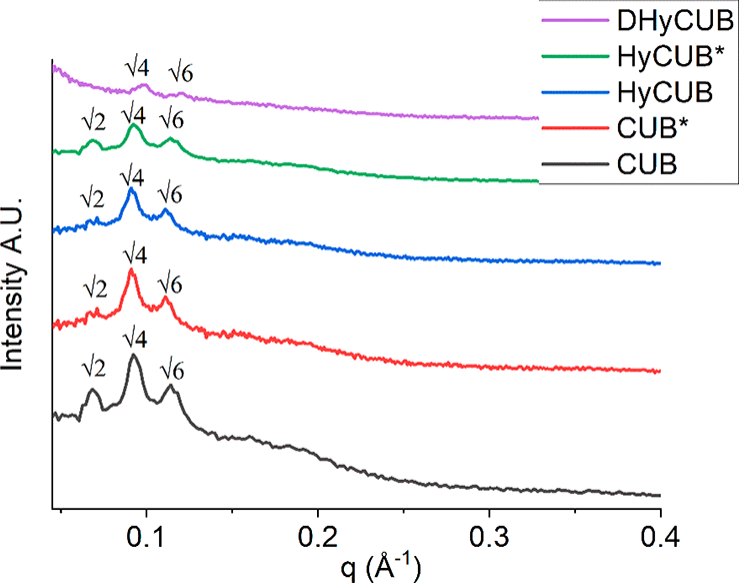
SAXS patterns of the investigated cubosomes.

DHyCUB, HyCUB*, HyCUB, CUB*, and CUB samples were analyzed by SAXS (Figure 2). For most of the samples, three characteristic scattering peaks corresponding to √2, √4, and √6 could be identified. Indeed, a SAXS diffractogram of sample DHyCUB shows only two peaks (Figure 2), very likely because of the decrease of the scattering intensity that makes the (weaker) √2 peak undetectable. In general, the bicontinuous cubic *Im*3*m* internal nanostructure was retained and confirmed for all the samples, with a calculated lattice parameter of 134 Å.

**Figure 3:**
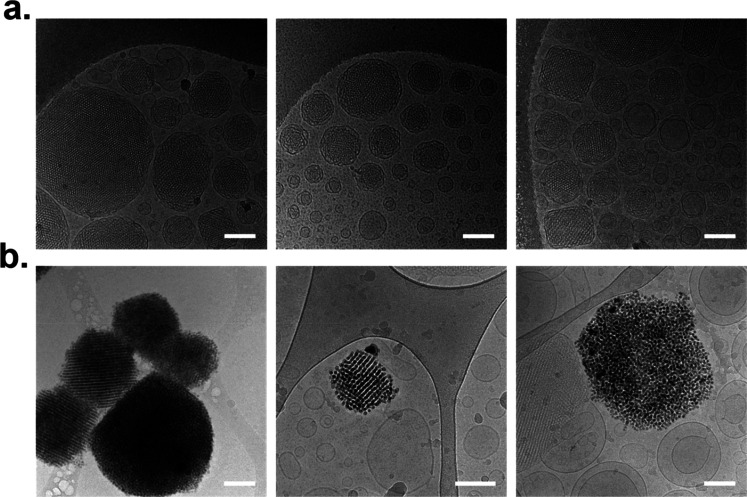
Cryo-TEM pictures of (a) CUB and (b) DHyCUB. Scale bar represents 100 nm.

Cryo-TEM was used to investigate the morphology of CUB and DHyCUB, as well as the localization of the UCNPs within the formulation (Figure 3). Cryo-TEM pictures of CUB formulation, shown in Figure 3a, are characterized by the simultaneous presence of quasi-spherical and squared-shaped nanoparticles showing an inner core presenting a dense, dark matrix alternate with bright spots, respectively corresponding to the cubosome lipid bilayer and the water channels. Small unilamellar vesicles can also be observed. Definitely, these images represent a cubosome formulation, as previously seen by cryo-transmission electron microscopy.^8,10^ Along with previously discussed SAXS experiments and stability tests, such observations prove that the LbL process here used to cover cubosomes with a CHIT/DNA/FA-CHIT shell can be considered as an efficient technique to stabilize cubosomes in water.

Cryo-TEM images of DHyCUB reported in Figure 3b give us a detailed image of the sample, from which an important feature of the formulation emerges; that is, all the UCNPs are embedded only within the cubosomes. Indeed, although some cubosomes not containing UCNPs can be found, none of the other nanoparticles (vesicles) visible in the pictures contain UCNPs. Importantly, in many cases the UCPNs appear arranged within the cubosomes according to their bicontinuous cubic original nanostructure. This finding can be inferred to the hydrophobic nature of the UCNPs and their consequent entrapment within the lipid bilayer.

### Optical Properties

**Figure 4:**
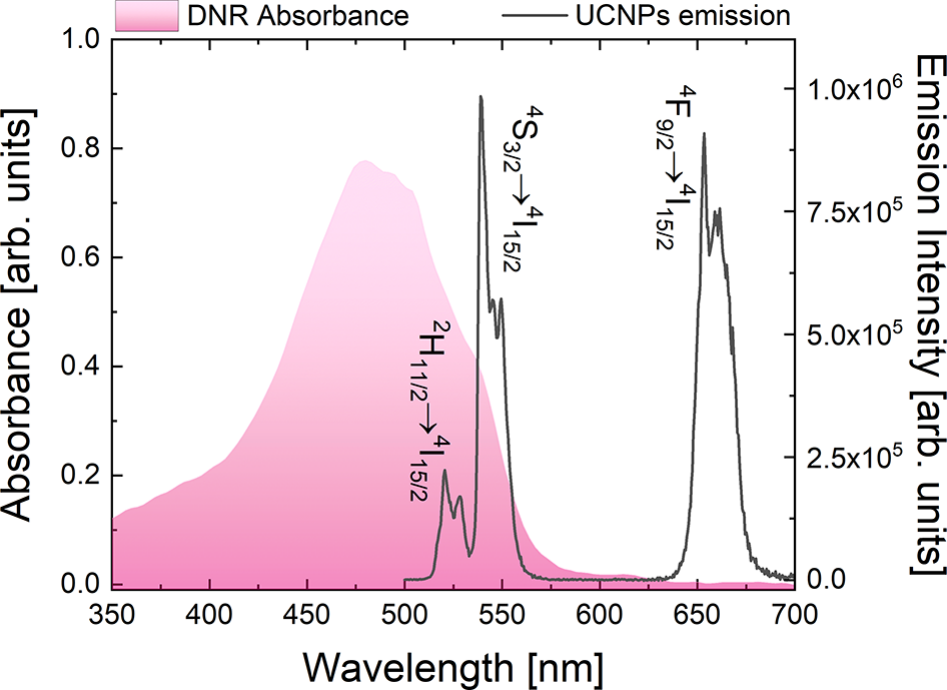
Up-conversion emission spectra (upon 980 nm laser diode excitation) of cubosomes loaded with NaYF_4_:Er^3+^,Yb^3+^ NPs compared with absorbance spectra of DNR molecules.

DHyCUB formulation was then optimized in terms of fluorophores mutual concentrations for the best optical performance. Doping of NaYF_4_ UCNPs (i.e., 2% of Er^3+^ ions and 20% of Yb^3+^ ions) was chosen to observe efficient visible UC emission after NIR 980 nm excitation (the emission lines peaking at approximately 520, 540, and 660 nm were attributed to the Er^3+^ ion ^2^H_11/2_ → ^4^I_15/2_, ^4^S_3/2_ → ^4^I_15/2_, and ^4^F_9/2_ → ^4^I_15/2_ transitions, respectively), while the absorption spectra of DNR were partly overlapping with the green region of the UCNPs spectrum (Figure 4). As it was already shown in previous studies,^22,23^ such conditions ensure the possibility to observe efficient energy transfer (static or dynamic) between nanocarrier components, thus allowing the triggering of the biological response after NIR 980 nm excitation. It was, however, necessary to search for the optimum DNR amount inside the cubosome. For this purpose, samples with a DNR concentration ranging from 0 up to 600 μM were prepared, and their physicochemical (i.e., *D*_H_, PdI and ζ-potential) and optical (i.e., UC emission spectra and lifetimes, UC Er^3+^ ions red-to-green emission ratio) parameters were investigated and reported in Table 2. *D*_H_, PdI, and ζ-potential values did not evidence any major changes upon loading of increasing amounts of DNR molecules, whereas the latter had a great influence on the optical properties of coloaded cubosomes. The reference sample, loaded only with NaYF_4_ UCNPs (HyCUB), showed intense visible emission (integral intensity up to ∼30 × 10^6^ au) with long luminescence lifetimes (τ = 223.9 μs for ^4^S_3/2_ → ^4^I_15/2_ transition and τ_1_ = 525.3 μs for ^4^F_9/2_ → ^4^I_15/2_ transition) is characteristic for encapsulated Er^3+^/Yb^3+^ doped NaYF_4_ UCNPs.^22^ Co-loading of DNR resulted in a 6-fold decrease of overall emission intensity (from ∼30 × 10^6^ down to 5 × 10^6^), however, keeping it still at a very high ×10^6^ range level. The intensity ratio between red and green (R/G) UC Er^3+^ ions emission bands was also calculated to verify if some energy transfers or reabsorption was present among coloaded components. On the basis of the spectra presented in Figure 4, showing the overlap between donor (UCNPs) emission spectra and acceptor (DNR molecules) absorption spectra, when the energy transfer (either static or dynamic) between UCNPs and DNR is observed, the green emission should decrease with respect to the red one that, on the contrary, should not be absorbed by the DNR molecules. As shown in Table 2, coloading of DNR molecules resulted in an increase of the R/G emission ratio, proving static or dynamic energy transfer^30^ between coloaded UCNPs and DNR molecules. This increase in the R/G emission ratio was higher in the sample coloaded with 200 μM DNR. Finally, to discriminate if static or dynamic energy transfer processes occurred, luminescence lifetimes (τ) for coloaded samples (Table 2) were also measured, recording a shortening of the τ values. Moreover, a change in the decay behavior from mono- to double-exponential upon coloading of DNR molecules was also evidenced. This finding can be related to the interaction between closely packed fluorophores within the cubosomes. Quenching of emission kinetics was stronger for the sample with 200 μM DNR added. Particularly, the τ value for green emission in this case was shortened from 223.9 μs measured for reference sample down to 55.5 μs, suggesting an efficient dynamic Förster Resonance Energy Transfer (FRET) between UCNPs and DNR molecules.^31^ Results of luminescence lifetime measurements converged with those of the R/G emission ratio parameter calculations, indicating the sample with coloaded 200 μM DNR as the most promising candidate for further biological studies. Figure S1 (see the Supporting Information) shows an additional comparison between the UC emission spectra (a) and green emission band luminescence kinetics (b) for the reference sample and the one with coloaded 200 μM DNR.

**Table 2:** Optimization Parameters of Cubosomes with Different Payloads^a^

sample	C_DNR_[μM]	EE_DNR_[%]	*D*_H_[nm]	PdI	ζ [mV]	integral luminescence intensity	red/green ratio	LT at 540 nm [μs]	LT at 660 nm [μs]
CUB	0		162 ± 7	0.184 ± 0.010	+13.5 ± 0.7				
HyCUB	0		167 ± 8	0.215 ± 0.012	+14.6 ± 0.8	29.7 × 10^6^	1.21	τ_1_ = 223.9 ± 8.2	τ_1_ = 525 ± 18
DHyCUB	200	90 ± 4	169 ± 8	0.253 ± 0.013	+14.4 ± 0.7	5.0 × 10^6^	1.48	τ_1_ = 55.5 ± 3.0	τ_1_ = 101.3 ± 5.6
								τ_2_ = 13.1 ± 1.9	τ_2_ = 28.2 ± 2.8
DHyCUB	400	86 ± 3	170 ± 8	0.284 ± 0.014	+14.0 ± 0.7	11.4 × 10^6^	1.15	τ_1_ = 181.0 ± 4.8	τ_1_ = 375 ± 11
								τ_2_ = 34.9 ± 2.5	
DHyCUB	600	83 ± 3	173 ± 9	0.273 ± 0.014	+14.7 ± 0.8	12.6 × 10^6^	1.42	τ_1_ = 200.6 ± 6.2	τ_1_ = 517 ± 15
								τ_2_ = 12.6 ± 1.3	τ_2_ = 27.3 ± 2.6

a CUB: cubosomes. HyCUB: hybrid cubosomes loaded with up-converting nanoparticles. DHyCUB: hybrid cubosomes loaded with up-converting nanoparticles and daunorubicin. *C*_DNR_: concentration of daunorubicin. EE_DNR_: encapsulation effcienty of daunorubicin. *D*_H_: hydrodynamic diameter measured by DLS. PdI: polidyspersity index measured by DLS. ζ: zeta potential. LT: luminescence lifetime.

### Cellular Uptake and NIR-Triggered Oxidation Efficiency

**Figure 5:**
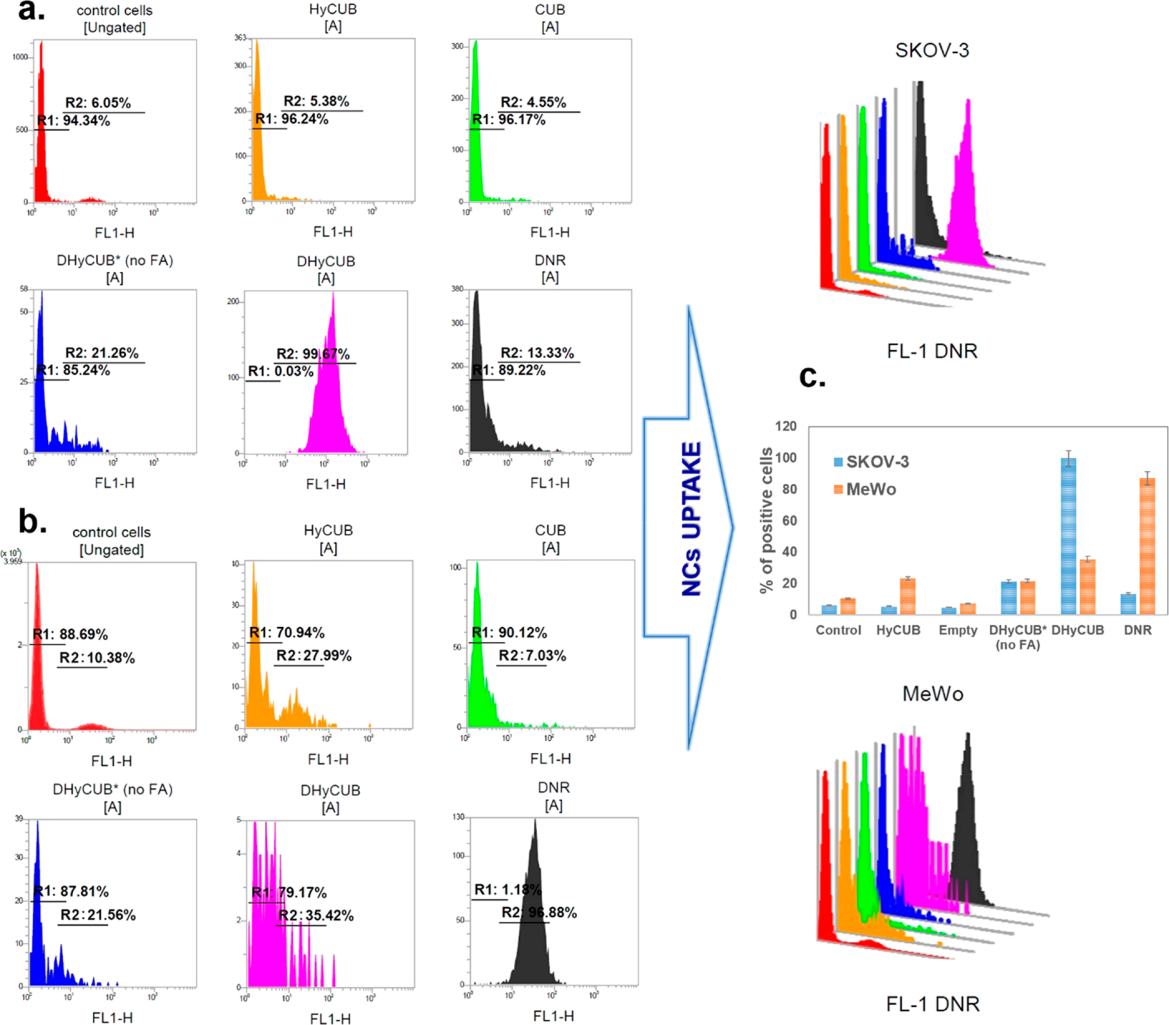
Flow cytometry histograms for the uptake of DHyCUB by (a) cancer human ovarian SKOV-3 and (b) MeWo melanoma cells after 24 h incubation at 37 °C, as well as the FACS uptake comparison for both cell lines (c). R corresponds to region of appropriate gate, where R1 are cells without fluorescent dye (DNR), and R2 corresponds to the region of cells, where DNR (2 μM as final drug concentration) was delivered/transported.

The first goal of this study was to obtain a biocompatible, theranostic formulation useful in oncology applications. Consequently, the cytotoxicity of DHyCUB, expressed as mitochondrial dehydrogenase activity (MTT assay), was investigated in different dilution conditions (from 1:50 to 1:500). The viability studies, estimated after 24 h of incubation of the cubosome formulation in dark conditions (Figure S2 in the Supporting Information) with the SKOV-3 and MeWo cancer cell lines, proved the cubosomes safety from 1:100 upward for both cancer cell lines in the absence of external irradiation. This screening study allowed the selection of the most favorable cubosome dilution (1:100), corresponding to the final 2 μM concentration of DNR for the following internalization experiment, aimed to evaluate the influence of the DNR and UCNPs coencapsulation and the cubosome surface engineering on the cellular uptake as provided by flow cytometry (FACS) (Figure 5). Importantly, the cellular uptake studies showed an almost 3 times improved efficacy in the active cargo (DNR + UCNPs) delivery by the FA-functionalized cubosomes to the human ovarian cancer cell (Figure 5a) line compared to delivery to the melanoma cells (Figure 5b). Interestingly, in the absence of FA functionalization (DHyCUB*, no FA), the uptake efficacy was comparable for both cell lines (Figure 5c). The superior performance of FA-functionalized cubosomes can be explained by the improved recognition by folate receptors (FRs) overexpressed on the SKOV-3 cell surface,^29^ resulting in enhanced targeting response for the treatment of that type of cancer cells. The beneficial effect of the FA functionalization of the cubosome surface on the active cargo delivery was additionally shown in some representative CLMS internalization images provided on SKOV-3 cell lines (Figure S3; see the SI).

**Figure 6:**
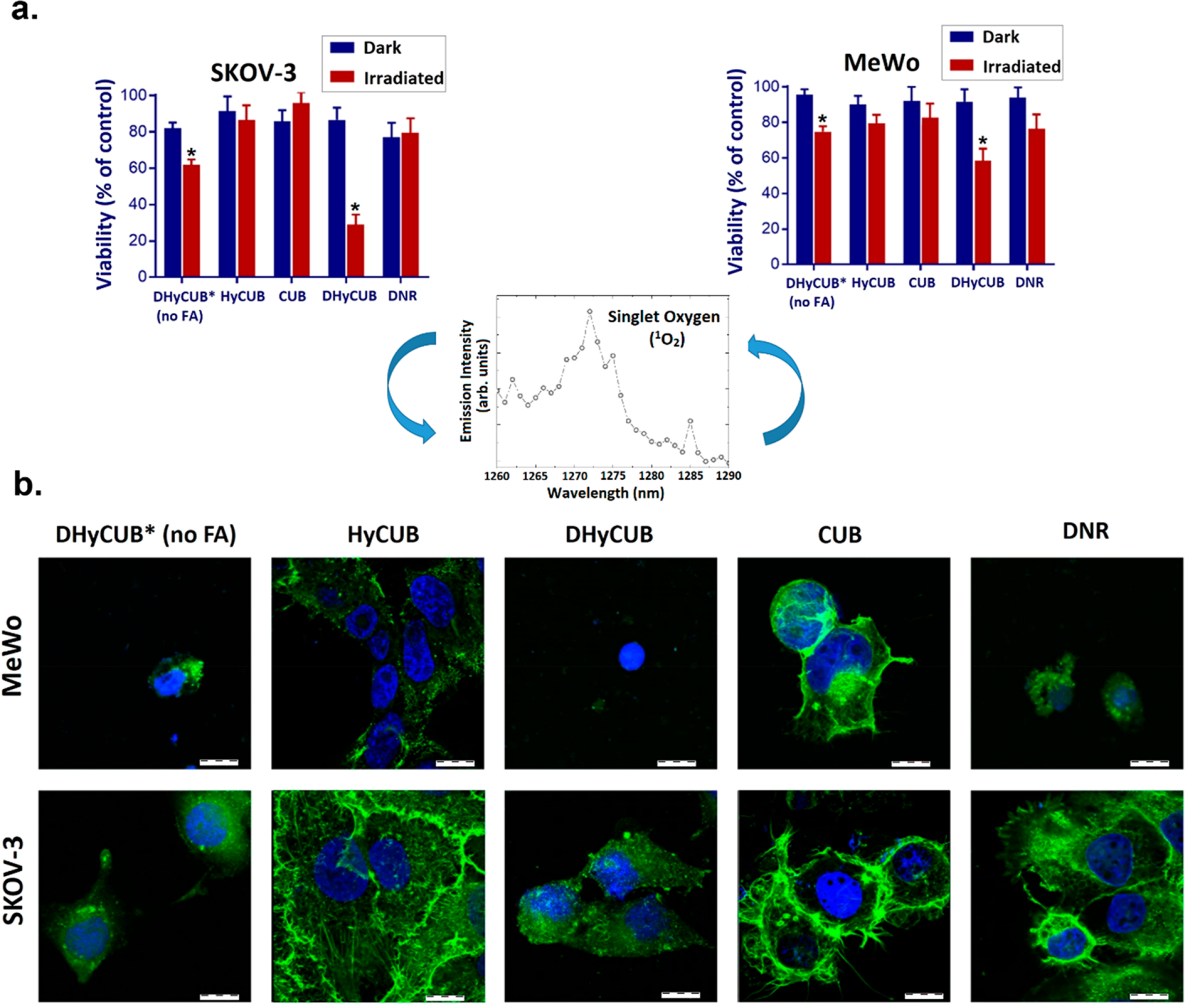
Photodynamic efficiency of DHyCUB (NaYF_4_:Er/Yb UCNPs coloaded with DNR in the final drug concentration of 2 μM, encapsulated in FA-functionalized cubosomes) expressed by (a) photocytotoxicity experiments (results are represented as percentage of control untreated cells, * statistical ANOVA analysis: *p* ≤ 0.05), and (b) oxidation of the filamentous actin (F-actin) cytoskeleton 24 h after NIR cell irradiation with a 980 nm laser diode compared to control results on human ovarian (SKOV-3) and melanoma (MeWo) cancer cells. Cells were irradiated using the following conditions: 6.2 W/cm^2^ for 5 min. Scale bar represents 100 μm. Inset: singlet oxygen (^1^O_2_) characteristic infrared emission measured for DHyCUB.

The key focus of the present investigation was on the NIR-triggered oxidation efficiency of the theranostic cubosomes. As it is shown in Figure 6, the improved photodynamic activity of the coencapsulated DNR and UCNPs upon SKOV-3 cells finds confirmation in the NIR-induced PDT, evaluated during two independent experiments, i.e., by measurements of photocytotoxicity using the MTT assay for assessing cell metabolic activity (Figure 6a), and by immunofluorescence method to evaluate the reorganization of cellular cytoskeleton after 980 nm laser irradiation (Figure 6b). The cancer cell exposure to NIR light followed by singlet oxygen generation (^1^O_2_) and 24 h incubation with the DHyCUB formulation significantly decreased the mitochondrial activity, leading to less dehydrogenase activity and resulting in the reduction of remaining viable cells in both SKOV-3 and MeWo cell lines (Figure 6a). However, such a photocytotoxic effect was found less important when both cancer cell lines were incubated with the formulation without the FA-CHIT as the outermost layer. Indeed, with a viability after irradiation of respectively 25% and 63% vs control, DHyCUB caused more than twice the decrease in cell survival in ovarian cancer cells (SKOV-3) with respect to DHyCUB*. When DHyCUB was incubated with melanoma cells (MeWo), its photocytotoxicity was found to be lower (58% vs control viability) than that observed in the SKOV-3 cell line but still higher than that induced by DHyCUB* (74% vs control viability). The stronger photocytotoxicity observed when DHyCUB is incubated with SKOV-3 cells can be easily related to the extremely high uptake of these FA-functionalized cubosomes by SKOV-3 cells detected during the FACS experiments (Figure 5). In the case of remaining control samples (empty or alternatively loaded with UCNPs or DNR), the photodynamic effect detected in the ovarian cells was not significant. For the controls in the melanoma cells, this effect was more noticeable, reaching the highest level for DNR of ca. 20% decrease in cell viability and ca. 15% difference from the same unexposed sample. The nonirradiated cells showed a very good level of the cells’ viability, indicating that the encapsulation process has a protective influence on the encapsulated payloads against the external cellular environment. The results of MTT photocytotoxicity studies applied to determine the dehydrogenase activity after NIR-induced activation were supported by the immunofluorescence study shown in Figure 6b. The examination, involving the cytoskeleton fluorescent staining after PDT action, revealed that the cytoskeleton is the most affected in the case of both cancer cells treated with encapsulated theranostic cargo (DHyCUB). A significant reorganization and destabilization of F-actin fibers was observed. Moreover, the cells’ shape was affected, and a strong shrinkage occurred after exposure to both variants of DHyCUB. HyCUB nanocarriers caused dispersal cytoskeleton organization (the fibers lost their continuity), and in the case of ovarian cancer, stress fibers appeared at the cells’ edges. Interestingly, DNR affected the cytoskeleton of melanoma cells, but not ovarian cancer cells, indicating resistance to this chemotherapeutic in free form and confirming our photocytotoxicity results presented in Figure 6a. Control cells and cells not exposed to irradiation showed unaffected cytoskeleton with properly tensioned actin fibers spanning the cytosol (results shown in Figure S4). Empty cubosomes, moreover, were found safe for both types of cells, while their morphology was not significantly changed. It deserves noticing that the photodynamic procedure exerted some influence on cell size and the cytoskeleton. Particularly, empty cubosomes combined with irradiation affected slightly F-actin fibers conditions, without any significant cell viability change. These results are in agreement with previous investigation on energy transfer between fluorophores coloaded in hybrid polymeric nanocarriers.^22,23^ The observations of reactive oxygen species release in SKOV-3 and MeWo cells show an increased ROS level after photodynamic reaction. Interestingly, various ROS release was in these two cell lines. Ovarian cancer cells reacted violently immediately after PDT (Figure S5 in the Supporting Information), while in the case of melanoma cells, ROS release increased with time (Figure S5). An increased ROS release in the first phase suggests that cells can undergo faster cell death. Undoubtedly, the evaluation of reactive oxygen species’ release demonstrated the highest activity of DHyCUB in melanoma and ovarian cancer cells. In other studies it was noted that the overproduction of reactive oxygen species can be a critical mediator of apoptotic or autophagic cell death.^34^

## Conclusions

In the present paper the structural design of lipid-based nanoparticles leading to FRET-activated cubosomes useful for NIR-induced photodynamic therapy upon human cancer cells was discussed. Particularly, lipid-based cubosomes were hybridized by coencapsulating inorganic up-converting Er^3+^ and Yb^3+^ codoped NaYF_4_ NPs, used as an energy harvesters and diagnostic agents. These nanoparticles were stabilized against flocculation by a shell consisting of alternate layers of chitosan, single strand DNA, and folic acid–chitosan conjugate (the outer layer). In addition, they were loaded with daunorubicin (DNR), applied as a chemotherapeutic drug of photosensitizing activity. The single strand DNA isolated from herring sperm was successfully deposited on the cubosomes surface, simultaneously acting as a negatively charged polyelectrolyte and model genetic material, while showing the versatility of the formulated nanoplatform and disclosing its potential in gene or vaccine therapy (e.g., against COVID-19). Internalization properties and NIR-induced photodynamic therapy were evaluated *in vitro* against human skin melanoma (MeWo) and ovarian (SKOV-3) cancer cells. Results evidenced an increased uptake of the theranostic cargo in ovarian cancer cells than in melanoma cells. This effect was enhanced in the case of FA-functionalized nanocarriers. The nanocarriers coloaded with DNR and UCNPs showed improved NIR-induced photodynamic activity, as confirmed by a decrease in both mitochondrial activity and the most affecting f-actin fibers of cytoskeleton. Similar results, but with higher photocytotoxic effect, were observed when FA-functionalized cubosomes were incubated with SKOV-3 cells. So far, only a few papers described the use of cubosomes as nanocarriers for encapsulation of inorganic, mostly magnetic, nanoparticles.^35,36^ Results here presented confirmed the potential of cubosomes as versatile and biocompatible nanosystem for theranostics cancer treatments.

## Experimental Section

### Chemicals and Reagents

The following chemicals for the preparation of theranostic cubosomes were purchased from Sigma-Aldrich: daunorubicin hydrochloride (DNR), Pluronic F108 (PF108), chitosan (CHIT, low molecular weight), DNA sodium salt from herring testes (DNA), Y_2_O_3_ (99.99%), Yb_2_O_3_ (99.99%), Er_2_O_3_ (99.99%), CF_3_COOH (reagent grade, 99%), oleic acid (technical grade, >93%), oleylamine (technical grade, 70%), folic acid (reagent grade, ≥97%). Monoolein (MO, 1-monooleoylglycerol, RYLO MG 19PHARMA, glycerol monooleate; 98.1 wt %) was a kind gift from Danisco A/S, DK-7200, Grinsted, Denmark. Other reagents and solvents were of commercial grade and were used as received. The water used for all experiments was doubly distilled and purified by means of a Milli-Q purification system (Millipore, Bedford, MA).

### Synthesis of Folic Acid–Chitosan Conjugate (FA-CHIT)

The folic acid–chitosan conjugate (FA-CHIT) was synthesized according to Scheme 1. A solution of folic acid (0.028 g, 6.4 × 10^–5^ mol) and 1-ethyl-3-(3-(dimethylamino)propyl)carbodiimide (EDC, 0.013 g, 6.4 × 10^–5^ mol) in anhydrous DMSO (20 mL) was prepared and stirred at room temperature until both EDC and FA were well-dissolved and mixed. The mixture was then slowly added to an acetic acid aqueous solution of chitosan (0.30 g, 2.5 × 10^–6^ mol) 0.5% (w/v) (0.1 M, pH 4.7) and stirred at room temperature in the dark for 16 h to let the FA conjugate onto the CHIT molecules. Then, the solution was brought to pH 9.0 by dripping with NaOH aqueous solution (1.0 M) and centrifuged at 2500 rpm to spin down the FA-CHIT. The precipitate was dialyzed first against phosphate buffered saline (PBS, pH 7.4) for 72 h and then 24 h against water. Finally, the FA-CHIT was isolated as a soft and fluffy product after freeze-drying.

### Synthesis of NaYF_4_:2%Er^3+^,20%Yb^3+^ Up-Converting Nanoparticles

About 8 nm in size lanthanide doped NaYF_4_ UCNPs^32,33^ were synthesized as follows. Lanthanide oxides (1 mmol, 78%Y_2_O_3_, 20%Yb_2_O_3_ and 2%Er_2_O_3_) and sodium hydroxide (2 mmol) were mixed and dissolved in 50% concentrated trifluoroacetic acid, followed by the residual water and acid evaporation. Afterward, the oleic acid (16 mL) and oleylamine (8 mL) were added, and the solution was mixed, heated up to ∼120 °C, and carefully degassed by alternating vacuum and inert gas conditions. For the synthesis the reaction mixture temperature was raised up to 275 °C and maintained for 30 min. After the mixture was cooled to room temperature, an excess of a mixture of methanol and acetone (2:1) was added, and the UCNPs were precipitated by centrifugation at 1000 rpm for 10 min. Washing with methanol was repeated two times in order to remove all the unattached ligands from the solution, and finally the UCNPs were dispersed in chloroform with the concentration of ∼95 mg/mL.

### Preparation of Hybrid, FA-Targeted, Theranostic Cubosomes

Hybrid, FA-targeted, theranostic cubosomes, loaded with NaYF_4_:Er^3+^,Yb^3+^ UCNPs and DNR, were prepared by using a two-step approach. In the first stage, MO-based cubosomes were prepared by dispersing MO and NaYF_4_:Er^3+^,Yb^3+^ UCNPs (initial concentration of 51 mg/mL) in a PF108 dichloromethane solution followed by 60 s of sonication with the help of an ultrasonic bath. Then, after the organic cosolvent evaporation under reduced pressure, an aqueous DNR solution was added dropwise under vigorous magnetic stirring at 40 °C for 1 h to prepare different formulations with final drug concentration of 200, 400, and 600 μM. The obtained aqueous suspension of cubosomes coloaded with NaYF_4_:Er^3+^,Yb^3+^ UCNPs and DNR, was finally sonicated by a tip sonicator (UP100H ultrasonic processor developed by Hielscher Teltow, Germany cycle 0.9, amplitude 90%) for 5 min and stored at room temperature overnight. In the second step, cubosomes were covered via the LbL saturation method,^26^ i.e., by selective adsorption of oppositely charged polyelectrolytes. CHIT, DNA, and the FA-CHIT were alternatively added onto the cubosomes surface to form a multilayered shell. A solution of DNA of concentration equal to 1 g/L was prepared by dissolving the polyion in a 0.015 M NaCl aqueous solution. To improve the solubility of CHIT, the polycation and its conjugate with FA were dissolved in 0.1 M CH_3_COOH. The optimal ratio of cubosomes/polyion (DNA, CHIT, and FA-CHIT) concentrations was determined by measuring the cubosomes ζ-potential and investigating their stability. In the final formulation the concentrations of the cubosome components were approximately: 1.238 wt % MO, 0.1125 wt % PF108, and 98.65 wt % water. After the preparation processes, the obtained samples were purified via the dialysis method according to the previously described protocol.^10^ The control (empty) cubosomes were prepared in a similar manner, but without the addition of the UCNPs and DNR.

### Cubosomes Size and ζ-Potential

The cubosomes average hydrodynamic diameter (*D*_H_) and polydispersity index (PdI) were determined by dynamic light scattering (DLS) using a Nano Series Zetasizer from Malvern Instruments, with a detection angle of 173° in optically homogeneous, squared polystyrene cells. All the measurements were performed at 25 °C. Each value was obtained as an average of three runs with at least 10 measurements. The ζ-potential of cubosomes was measured by the microelectrophoretic method by the Malvern Zetasizer Nano ZS apparatus. Each value was obtained as an average of three subsequent runs of the instrument, with at least 20 measurements. All samples were diluted with double distilled water in the ratio 1:50 before *D*_H_ and ζ-potential measurements. The DTS (Nano) program was used for data evaluation.

### Cubosomes Nanostructure (Small-Angle X-ray Scattering, SAXS)

SAXS measurements were performed to identify the internal nanostructure of the lipidic nanoparticles. The samples were analyzed with a MicroMax-002 with a microfocused beam, with a voltage of 45 kV, a filament current of 0.88 mA, and Ni-filtered Cu Ka radiation (λ = 1.5418 Å), collimated by three pinhole (0.4, 0.3, and 0.8 mm) collimators. The data were collected with a two-dimensional argon-filled Triton detector with an effective scattering-vector range of 0.03–0.45 Å^–1^. The samples were loaded in quartz capillaries with a thickness of 1.5 mm and sealed. All the samples were analyzed at 25 °C and under a vacuum.

### Cubosomes Morphology (Cryogenic Transmission Electron Microscopy, cryo-TEM)

Lacey carbon-coated 300-mesh copper grids (EMS, Hatfield, USA) were glow discharged (Emitech K100X, Quorum Technologies Ltd., Laughton, GB) for 30 s. An aliquot (4 μL) of sample was applied onto the grids in a Vitrobot Mark II (Thermo Fisher Scientific (TFS), Waltham, USA), and the excess of the sample was removed by controlled blotting at 95% humidity. A mixture of liquid ethane/propane was used for sample vitrification. The grids were then transferred on a Gatan cryo-holder (AMETEK, Pleasanton, USA) into a Tecnai F20 cryoTEM (Thermo Fisher Scientific (TFS), Waltham USA) and kept at −180 °C during observation. Micrographs were recorded under low dose conditions (<20 e^–^/A^2^) using a Falcon II 4K camera (TFS, Waltham, USA) operating the microscope at 200 kV acceleration voltage in bright field mode.

### DNR Encapsulation Efficiency (UV–Vis Spectroscopy)

To evaluate the DNR encapsulation efficiency (EE%) in the cubosome formulation, UV–vis absorbance measurements were performed after dialysis of the nonencapsulated drug, using a previously described purification method.^10^ Then, the cubosomes were disrupted after solubilization with a THF/water 3:1 mixture. The UV–vis measurements were performed using a Metertech SP8001 spectrophotometer with 1 cm path thermostated quartz cell and reading the absorption maxima at λ_DNR_ = 495 nm. The drug encapsulation efficacy was quantified as follows: EE% = (weight of the DNR in cubosomes)/(weight of the added DNR) × 100.

### Optical Properties and NIR-Activated ^1^O_2_ Generation

The UC emission spectra and luminescence lifetimes of water suspensions of cubosomes containing only NaYF_4_:Er^3+^,Yb^3+^ UCNPs and different ratios of coencapsulated NaYF_4_:Er^3+^,Yb^3+^ UCNPs and DNR were measured with an Edinburgh Instruments FLS 980 spectrofluorometer under a 980 nm fractionated laser diode (8 W, Spectra Laser, Poland). The emission decay curves were measured at 540 nm (^2^H_11/2_ + ^4^S_3/2_ → ^4^I_15/2_ Er^3+^ ions transition) and 670 nm (^4^F_9/2_ → ^4^I_15/2_ Er^3+^ ions transition) and fitted with a single exponential model. The ability of the encapsulated hybrid cargo to NIR-activated singlet oxygen (^1^O_2_) generation was evaluated by the direct measurements of ^1^O_2_ characteristic emission ∼1270 nm. Those measurements were performed for the dried sample. For that purpose, we used a home-built setup constructed from Omni-λ 300 Zolix spectrograph with a mounted cooled infrared detector module (Hamamatsu), and excitation from a fractionated fiber coupled 980 nm laser system (Optoelectronics Tech). Signals from the excitation source and the detector were fed to Lock-in Amplifier (SciTec Instrument), and spectra were collected by the dedicated Zolix software.

### Cell Culture for Biological Analysis and MTT Assay for Cytotoxicity

Biological studies were performed on human ovarian carcinoma cells resistant to diphtheria toxin, cisplatin, and Adriamycin (SKOV-3) and human melanoma granular fibroblasts (MeWo). The cancer cells were purchased from ATCC (SKOV-3) and ECACC General Cell Collection (MeWo) and cultured according to conditions previously described.^10,22^ For the experiments, cells were washed with sterile PBS (Phosphate Buffered Saline, LabEmpire, Poland) and then removed by trypsinization (Trypsin–EDTA, Sigma–Aldrich, USA). Cells were maintained in a humidified atmosphere at 37 °C and 5% CO_2_. The *in vitro* cytotoxicity evaluation of cubosomes loaded with NaYF_4_:Er^3+^,Yb^3+^ UCNPs and DNR was performed using a MTT reduction assay, after 24 h of the cell treatment according to the manufacturer’s protocol.^10^ The selected sample coloaded with 200 μM of DNR was measured at four different dilutions (1:50, 1:100, 1:200, 1:500), corresponding to the final 4–0.4 μM concentrations of DNR. Mitochondrial function was expressed as a percentage of viable cells under treatment relative to untreated control cells, followed by determination of the absorbance using a multiwell scanning spectrophotometer at 570 nm (EnSpire Perkin–Elmer, Poland).

### Cellular Uptake and FA Competition (FACS Protocol)

Flow cytometric analysis was performed for the assessment of the ability to internalize and uptake of the hybrid theranostic cubosomes in SKOV-3 and MeWo cells. Cells were seeded on 24-well plates at density of 4 × 10^4^, and left to attach overnight. The suspension of cubosomes was added with final DNR concentration equal to 2.0 × 10^–6^ M (2 μM), and cells were incubated for 24 h at 37 °C in a humidified atmosphere containing 5% CO_2_. Then cells were washed in PBS (without calcium and magnesium ions, IITD, Poland), trypsynized, and resuspended in 0.5 mL of PBS. Flow cytometric analysis was performed on a Cube 6 flow cytometer (Sysmex, Poland). The fluorescence of DNR was measured with a FL-5-H detector. In the analysis were included 10 000 events from each sample. Data were collected and analyzed by CyView software (Sysmex, Poland).

### NIR-Induced Photodynamic Reaction *in Vitro* and Immunofluorescence Protocol for Confocal Laser Scanning Microscopy (CLSM)

NIR-induced photodynamic reaction (PDR) was evaluated in two different ways, i.e., by measurements of photocytotoxicity using the MTT assay and by the immunofluorescence method to evaluate the reorganization of cellular cytoskeleton organization after 980 nm laser irradiation, both applied for NIR-activated theranostic cubosomes loaded in the SKOV-3 and MeWo cells. For both the experiments the most favorable cubosome dilution (1:100), corresponding to the final 2 μM concentration of DNR was used. For the photocytotoxicity experiments, the cells cultured for 24 h were treated with the nanocarriers and incubated for 24 h at 37 °C in a humidified atmosphere containing 5% CO_2_ followed by the replacement of cell culture medium with a fresh one. Then, the systems were irradiated with the 980 nm laser (Spectra Laser, Poland) diode with 6.2 W/cm^2^ light intensity for 5 min, at NIR-irradiation conditions fixed according to our previous study.^23^ After irradiation, the cells were incubated 24 h and MTT assay was performed according to the manufacturer’s protocol. For immunocytotoxicity experiments, SKOV-3 and MeWo cells were grown on coverslips for 24 h, and then treated with the coencapsulated NaYF_4_:Er^3+^,Yb^3+^ UCNPs and DNR. After incubation, samples were irradiated with conditions similar to those of the photocytotoxicity experiments *in vitro*, i.e., for 5 min using the 980 nm laser diode with 6.2 W/cm^2^ light intensity. Then they were fixed with 4% paraformaldehyde (PFA) in PBS, permeabilized with 0.5% Triton X-100 in PBS (v/v) (for 5 min at room temperature) and blocked with 1% bovine serum albumin (BSA) in PBS (for 30 min at room temperature). All wash steps were performed with PBS. The following antibodies were used: monoclonal F-actin antibody produced in mouse (2 μg/mL; overnight incubation at 4 °C; 1:100; Sigma-Aldrich) selected as the primary antibody for F-actin identification (the most crucial protein responsible for the cell shape and morphology) and the secondary antibody AlexaFluor488 conjugated (for 1 h at 37 °C; 1:50; Sigma-Aldrich). DNA was stained with DAPI (4,6-diamidino-2-phenylindole; 0.2 μg/mL). Cells were mounted in fluorescence mounting medium (DAKO). CLSM imaging was performed using an Olympus FluoView FV10i confocal laser scanning microscope (Olympus, Poland). Cell nuclei were detected by 405 nm excitation and 450/50 emission laser, F-actin was detected by 473 and 525 nm emission lasers. Images were recorded by a Plan-Apochromat 60× oil-immersion objective.

### Statistical Analysis

Results of the *in vitro* experiments were presented as means ± standard deviation (SD) values for minimum *n* = 3 and compared by two-way ANOVA for multiple comparisons and α = 0.05. Comparisons of samples exhibiting *P* values ≤0.05 were considered as statistically significant. Results were analyzed with commercial software (GraphPad Prism 7.0).

## Supporting Information Available

Up-conversion emission spectra and green emission band luminescence kinetics for the reference sample and the one with coloaded 200 μM DNR; cytotoxicity studies of DHyCUB upon human ovarian (SKOV-3) and melanoma (MeWo) cancer cells; intracellular distribution of DNR delivered with cubosomes to SKOV-3 cells; bioimaging of F-actin cytoskeleton of nonirradiated SKOV-3 and MeWo cells treated by the studied cubosomes and nontreated control cells; reactive oxygen species (ROS) release in both the studied cancer cell lines after NIR cell irradiation of the studied samples compared to the controls (PDF)
