# Supplementary material for: Hybrid Theranostic Cubosomes for Efficient NIR-Induced Photodynamic Therapy
Source: ACS Nano. 2022 Mar 25;16(4):5427–38. doi: 10.1021/acsnano.1c09367 (PMC9047672; doi:10.1021/acsnano.1c09367)
Supplement: Supplementary file 1 — nn1c09367_si_001.pdf [file nn1c09367_si_001.pdf]

Hybrid theranostic cubosomes for efficient NIR-induced photodynamic therapy

Urszula Bazylińska,[1] Dominika Wawrzyńczyk,[2] Julita Kulbacka,[3] Giacomo Picci,[4] Livia Salvati Manni,[5,6a] Stephan Handschin,[6b] Marco Fornasier,[4,7] Claudia Caltagirone,[4] Raffaele Mezzenga,[6a,6c]* Sergio Murgia[8]**

[1]Department of Physical and Quantum Chemistry, Faculty of Chemistry, Wroclaw University of Science and Technology, Wybrzeze Wyspianskiego 27, 50-370, Wroclaw, Poland.

[2]Advanced Materials Engineering and Modelling Group, Faculty of Chemistry, Wroclaw University of Science and Technology, Wybrzeze Wyspianskiego 27, 50-370, Wroclaw, Poland

[3]Department of Molecular and Cellular Biology, Faculty of Pharmacy, Wroclaw Medical University, Borowska 211 A, 50-556 Wroclaw, Poland

[4]Department of Chemical and Geological Sciences, University of Cagliari and CSGI, s.s. 554 bivio Sestu, I-09042 Monserrato (CA), Italy

[5]School of Medical Sciences, School of Chemistry and University of Sydney Nano Institute, The University of Sydney, NSW 2006, Australia

[6a]ETH Zurich Department of Health Sciences & Technology, Schmelzbergstrasse 9, Zurich, 8093, Switzerland

[6b]ETH Zurich Scientific Center for Optical and Electron Microscopy (ScopeM), Otto-Stern-Weg 3, Zurich, 8093, Switzerland

[6c]ETH Zurich Department of Materials, Wolfgang-Pauli-Strasse 10, Zurich, 8093, Switzerland

[7]Department of Chemistry, Lund University, SE-22100 Lund, Sweden

[8]Department of Life and Environmental Sciences, University of Cagliari and CSGI, via Ospedale 72, I-09124 Cagliari, Italy

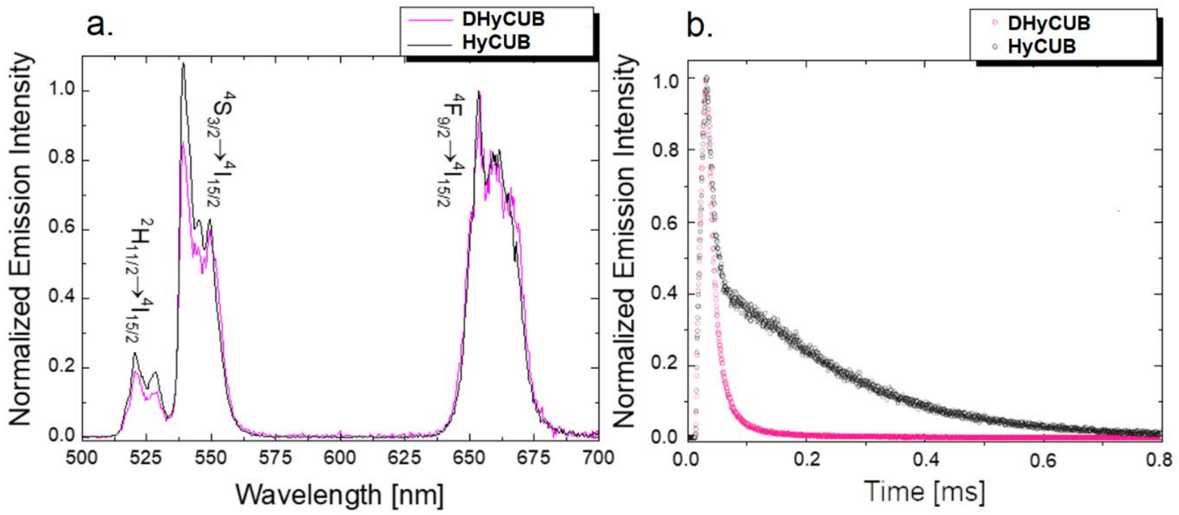

Figure S1. Up-conversion emission spectra (upon 980 nm laser diode excitation) of cubosomes loaded with $NaYF_4$:Er^{3+},Yb^{3+} UCNPs only (HyCUB, black line) and co-loaded with $NaYF_4$:Er^{3+},Yb^{3+} NPs and 200 μM of DNR molecules (DHyCUB, pink) (a), luminescence lifetime decay curves of Er^{3+} ions $^2H_{11/2}$ + $^4S_{3/2}$ energy level measured for sample loaded with HyCUB (black line) and DHyCUB (pink) (b).

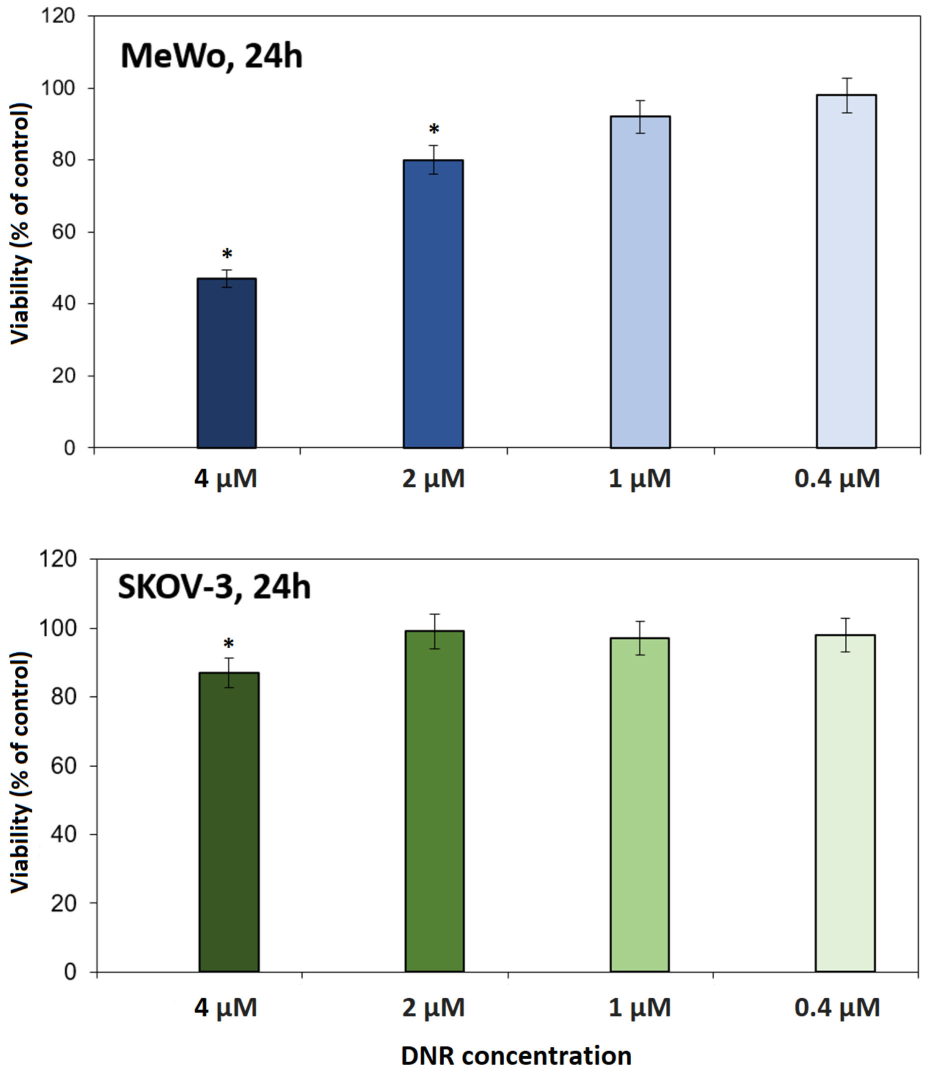

Figure S2. Cytotoxicity studies of DHyCUB (initial 200 µM concentration of DNR) estimated after 24 h of incubation of the cubosome formulation in dark condition. Data represented as means ±SD for minimum n = 3, where * $P \leq 0.05$ was judged as statistically significant.

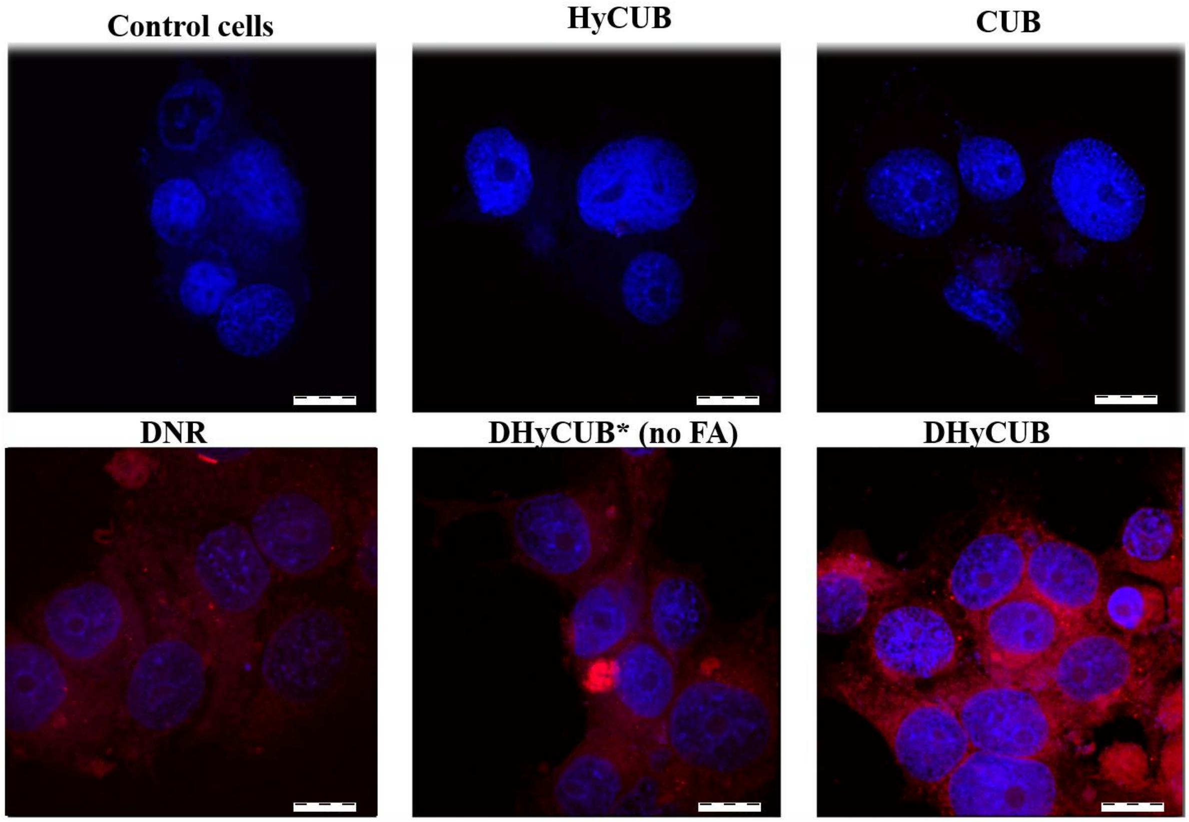

Figure S3. Intracellular distribution of DNR (in the final drug concentration of 2 μM) delivered with FA-functionalized cubosomes (DHyCUB) to the human resistant ovarian cancer (SKOV-3) cells after 24 h incubation. Nuclei stained with DAPI (nuclear staining) in blue and DNR signal in red. Scale bar represents 100 μm.

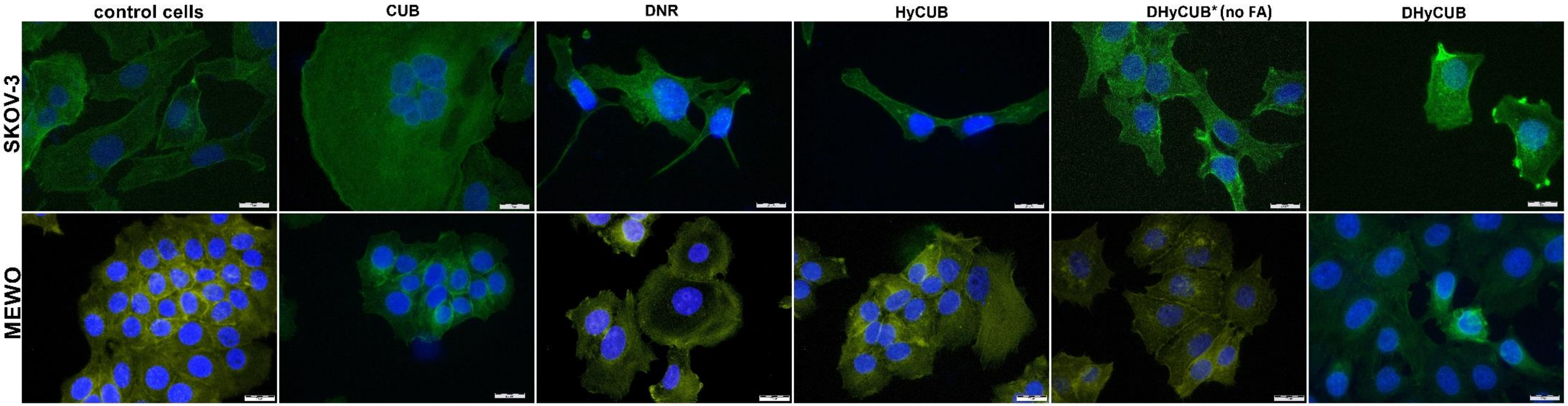

Figure S4. Bioimaging of filamentous actin (F-actin) cytoskeleton of non-irradiated human ovarian (SKOV-3) and melanoma (MeWo) cancer cells treated by the studied cubosomes and non-treated control cells. Scale bar represents 25 μm.

Reactive oxygen species detection by DCF assay

ROS production in both cancer cell lines after photodynamic reaction was evaluated by DCFDA / H_2DCFDA - Cellular ROS Assay Kit (ab113851, Abcam, Poland). Firstly, cells were cultured on black 96-well plates with the flat transparent bottom (Perkin Elmer, Poland) in concentration 25×10^3 per well. Photodynamic experiments with cubosomes were performed as previously described. DCF (2',7'-dichlorofluorescein) protocol for adherent cells was used. All buffers and DCFDA (2’,7’-dichlorofluorescein diacetate) solution were freshly prepared before usage. Cells were irradiated with a 980 nm laser diode (Spectra Laser, Poland) with 6.2 W/cm^2 light intensity for 5 min (the same conditions used for photocytotoxicity and immunofluorescence experiments shown in Figure 6). ROS release was measured after 30, 90 min, and 4 h post NIR irradiation. The fluorescent signal was measured at the excitation wavelength of 495 nm and emission wavelength of 530 nm by a multiwell reader Glomax (Glomax, Promega, GmbH, Germany). Results were normalized to the untreated control.

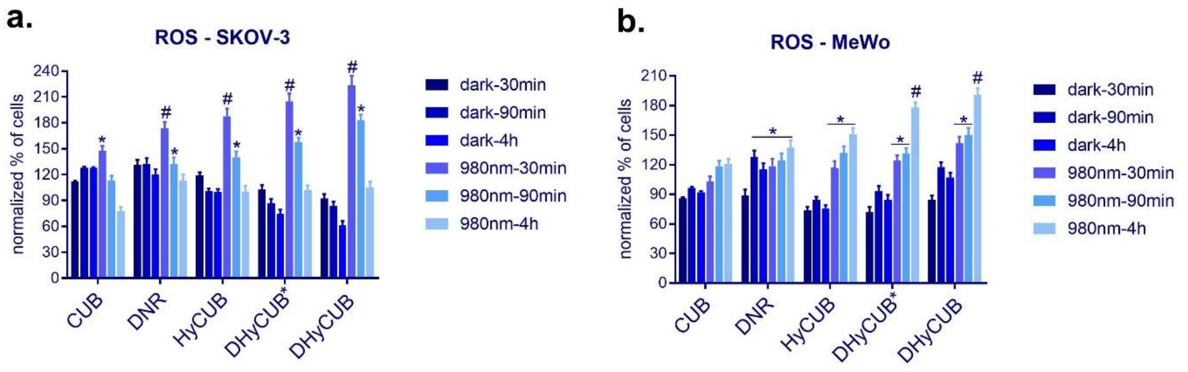

Figure S5. Reactive oxygen species (ROS) release in (**a**) SKOV-3 and (**b**) MeWo cells, after NIRcell irradiation by 980 nm laser diode of NaYF4:Er/Yb UCNPs co-loaded with DNR (in the final drug concentration of 2 μM) in FA-functionalized cubosomes (DHyCUB), compared to the controls. **p≤0.05; # p≤0.005.*
